# Comparison of Antimicrobial Effects of Triple Antibiotic Paste and Calcium Hydroxide Mixed with 2% Chlorhexidine as Intracanal Medicaments Against Enterococcus faecalis Biofilm

**Published:** 2018-05

**Authors:** Sholeh Ghabraei, Behnam Bolhari, Mohammad Marvi Sabbagh, Mahsa Sobhi Afshar

**Affiliations:** 1 Assistant Professor, Department of Endodontics, School of Dentistry, Tehran University of Medical Sciences, Tehran, Iran; 2 Associate Professor, Laser Research Center of Dentistry, Dentistry Research Institute, Tehran University of Medical Sciences, Tehran, Iran; Department of Endodontics, School of Dentistry, Tehran University of Medical Sciences, Tehran, Iran; 3 Dentist, Private Practice, Tehran, Iran; 4 Postgraduate Student, Department of Endodontics, School of Dentistry, Tehran University of Medical Sciences, Tehran, Iran

**Keywords:** Doxycycline, Metronidazole, Ciprofloxacin, Calcium Hydroxide, Chlorhexidine, Enterococcus Faecalis

## Abstract

**Objectives:**

The purpose of this in-vitro study was to determine and compare the shortest period needed for a triple antibiotic paste (TAP) and calcium hydroxide (Ca(OH)_2_) plus 2% chlorhexidine (CHX) to eradicate the biofilm of Enterococcus *faecalis* (EF) from the root canal system.

**Materials and Methods:**

Sixty-five extracted single-rooted human teeth with straight root canals were selected. The crowns were cut from the cementoenamel junction (CEJ), and canal preparations were done by step-back technique. The smear layer was removed by 17% ethylenediaminetetraacetic acid (EDTA) and 5.25% sodium hypochlorite (NaOCl). Afterwards, the samples were sterilized with gamma ray and were placed inside microtubes for one week. During this week, the teeth were infected with EF. Then, a TAP and Ca(OH)_2_ mixed with 2% CHX were inserted into the canals. The roots were cut longitudinally, and dentin chips were collected from the apical part of the roots by a round bur to the depth of 400 μm. The vital bacterial load was assessed by counting the numbers of colony-forming units (CFUs).

**Results:**

The paste of Ca(OH)_2_ mixed with 2% CHX was able to eradicate the EF biofilm in three days. The TAP was able to eradicate the biofilm of EF in seven days.

**Conclusions:**

It seems that Ca(OH)_2_ mixed with 2% CHX is more potent than the TAP against EF biofilm.

## INTRODUCTION

Enterococcus *faecalis* (EF) is found in 4–40% of primary endodontic infections and it is one of the bacteria mainly involved in treatment-resistant periradicular lesions [1]. This bacterium is one of the most frequently isolated species from failed root canal treatments and resistant infections [1,2]. *EF* shows resistance to some antimicrobial agents such as calcium hydroxide (Ca(OH)_2_ with a high pH [2]. This bacterium can also be found in the oral cavity; therefore, it can enter into the root canal before, during, and after root canal therapy and can cause reinfection [2]. There are several studies which have reported the presence of *EF* in the root canal system after endodontic treatments [3,4]. This microorganism has the ability to suppress the lymphocytes present in the root canal system, leading to endodontic failure [5]. *EF* is also able to form a biofilm which can decompose dentin in a food-free environment and can penetrate into dentinal tubules [6]. Although mechanical cleansing with detergents during endodontic treatments reduce microorganisms of the root canal system; it is recommended to use intracanal medicaments (ICMs) between treatment sessions in some conditions [7–9]. This does not indicate the necessity of their use in the treatment; however, they can lead to a better prognosis in the treatment of secondary or resistant infections that do not respond well to primary endodontic treatments. Ca(OH)_2_ is the most common ICM that is widely used in endodontic treatments and has antimicrobial properties due to its alkaline pH [10]. The effect of this substance on the biofilm of *EF* bacterium is controversial [10–15]. Chlorhexidine (CHX), which is commonly used as an irrigant during endodontic treatments, has a strong antimicrobial effect; therefore, it is also used as an ICM, either alone (gel with the concentration of 2%) or in combination with other drugs (in liquid form) [16]. Different studies have shown that the use of 2% CHX gel alone, in comparison with Ca(OH)_2_ with or without 2% CHX, has greater antibacterial effects in root canals [17–20]. One of the substances that have been found to have good antibacterial properties as an ICM is the triple antibiotic paste (TAP). This paste is prepared from a combination of three antibiotics (Minocycline, Metronidazole, and Ciprofloxacin) with normal saline, and as an ICM, it can remove bacteria even from the very deep areas of root canals [21]. Studies on regenerative treatments are generally case-reports, and there is no consensus on the duration of insertion of the mentioned materials into the canal.

Haapasalo and Orstavik [22] reported that Ca(OH)_2_ cannot remove *EF* even from the superficial layers of dentinal tubules. Siqueira and de Uzeda [23] also came up with the same result in their study.

Chai et al [24] investigated the effect of Ca(OH)_2_ and a series of antibiotics on the *EF* biofilm that was cultured on membrane filters in a laboratory study. It was concluded that Erythromycin, Oxytetracycline, and Ca(OH)_2_ completely eliminated the *EF* biofilm.

Gomes et al [17] showed that the use of Ca(OH)_2_ in combination with saline cannot play a role in reducing the *EF*, but if this paste is combined with 2% CHX, it will have an antimicrobial activity for up to 7 days, and after that time, its antimicrobial effect will be reduced. Evans et al [12] and Schafer and Bossmann [19] also confirmed this conclusion.

Taneja et al [25], in evaluating a case of a young permanent tooth with a necrotic pulp and a large periradicular lesion that had not been healed after intracanal Ca(OH)_2_ therapy, observed that the periradicular lesion completely resolved, the apical part of the root continued to grow, and the root apex was completely closed by using a TAP in the canal.

Adl et al [26] concluded that a TAP in combination with 2% CHX or normal saline was more effective than Ca(OH)_2_ paste in combination with 2% CHX or saline in the elimination of *EF*. They also concluded that the greatest antibacterial effect among the components of a TAP is related to Minocycline. Since these two agents are both used as ICMs for the treatment of resistant infections and in regenerative treatments, we tried to compare a TAP and Ca(OH)_2_ plus 2% CHX in the complete elimination of the *EF* biofilm in a laboratory condition, and we also tried to determine the shortest time required by each of these two substances to reach the desired function, which is rendering a root canal system free of EF biofilm.

## MATERIALS AND METHODS

### Pilot study:

A pilot study was conducted, which its purpose, similar to that of the original study, was to determine the shortest time required by the ICM to completely remove the *EF* biofilm from the prepared specimens. Therefore, 24 extracted human single-rooted teeth that were sterilized with gamma rays (40 kGy) were immersed in a suspension of *EF* (ATCC 29212) in Trypticase Soy Broth (TSB; Merck, Darmstadt, Germany) for seven days. The culture medium was changed every other day. After one week of incubation, four teeth were sampled, and the colony-forming unit (CFU)/ml was calculated. The results are presented in Table 1.

**Table 1.** Colony-forming unit (CFU)/ml of the four samples after a week of incubation with Enterococcus faecalis (EF) suspension

**Sample number**	**Total weight of dentin chips (g)**	**CFU/ml**
1	0.007	4.2×10^5^
2	0.007	3.8×10^5^
3	0.007	4.4×10^5^
4	0.007	3.2×10^5^
**Mean CFU/ml=3.9×10^5^**		

Twenty teeth were divided into two groups of 10: the first group received the TAP, and the second group received Ca(OH)_2_/2% CHX as the ICM. Then, the apical and coronal parts of the teeth were sealed, and the teeth were kept at 37°C. At 12 hours, 1 day, 3 days, 5 days, and 7 days after the insertion of the ICMs in the canals, two teeth from each group were chosen, and after removing the coronal seal, the root canal of each tooth was irrigated with 1cc of sterile saline and was dried by sterile paper cones (Ariadent Co., Tehran, Iran).

Considering the importance of cleansing the apical area of the root canal during endodontic treatments, and difficult access as well as a higher load of bacterial biofilm in this region, the mid-apical portion was used to obtain dentinal chips [27]. A #04 round carbide bur (Teeskavan Co., Tehran, Iran) was used for this purpose. Serial dilution method with a 10-fold dilution was used, and the total CFU/ml was calculated. The results are presented in Table 2.

**Table 2.** Colony-forming unit (CFU)/ml of each study group at different time intervals in the pilot study

**Time**	**Tooth number**	**TAP**	**Ca(OH)_2_/2% CHX**
**12 hours**	1	2.16 × 10^5^	1.52 × 10^5^
	2	2.02 × 10^5^	1.24 × 10^5^
**1 day**	1	1.14 × 10^5^	4.4 × 10^4^
	2	1.24 × 10^5^	3.2 × 10^4^
**3 days**	1	3.4 × 10^4^	0
	2	2.2 × 10^4^	0
**5 days**	1	9.8 × 10^3^	0
	2	6.6 × 10^3^	0
**7 days**	1	0	0
	2	0	0

TAP=Triple antibiotic paste, Ca(OH)_2_=Calcium hydroxide, CHX=Chlorhexidine

According to Table 2, after seven days for the TAP and three days for Ca(OH)_2_/2% CHX paste, the *EF* concentration in the dentin chips reached zero. For this reason, day seven and day three were selected for sampling the two groups of 1 and 2, respectively, in the main study.

### Main study

The main experimental study was performed on 65 recently extracted single-rooted teeth. Teeth with calcified canals, root caries, or open apices which could cause an error in the study were excluded. To prevent dehydration, the teeth were stored in 0.9% normal saline from the time they were extracted. For an easier access to the apical third of root canals, a diamond disk (DiaDent, Maribor, Slovenia) was used to cut the crown from the cementoenamel junction (CEJ) and to standardize the working length (WL) of all the samples. After WL determination, preparation and shaping of the root canals were manually done with K-type stainless steel files (Mani, Tochigi, Japan) and via the step-back technique to the apical size of 35. To remove the smear layer from root canal walls, 5.25% sodium hypochlorite (NaOCl, Yekta, PakNam Co., Tehran, Iran) and 17% ethylenediaminetetraacetic acid (EDTA, Ariadent, Asia Chemi Teb Co., Tehran, Iran) were respectively used for 1 minute, and 0.9% normal saline was also introduced into the canals between the two previous irrigants. Normal saline was also used as the final rinse.

During the process of preparing and shaping of root canals, the prepared teeth were placed in microtubes containing 1.5 ml of fresh distilled water. Tooth sterilization was performed with gamma rays with a dose of 40 kGy for 3 hours and 45 minutes (Atomic Energy Organization, Department of Radiation, North AmirAbad, Tehran, Iran) [28].

In the microbiology laboratory, dental sterilization was evaluated: three teeth were selected randomly from the sterilized teeth and were placed in the culture medium. No growth of any bacteria was observed in the culture medium.

**Fig. 1:**
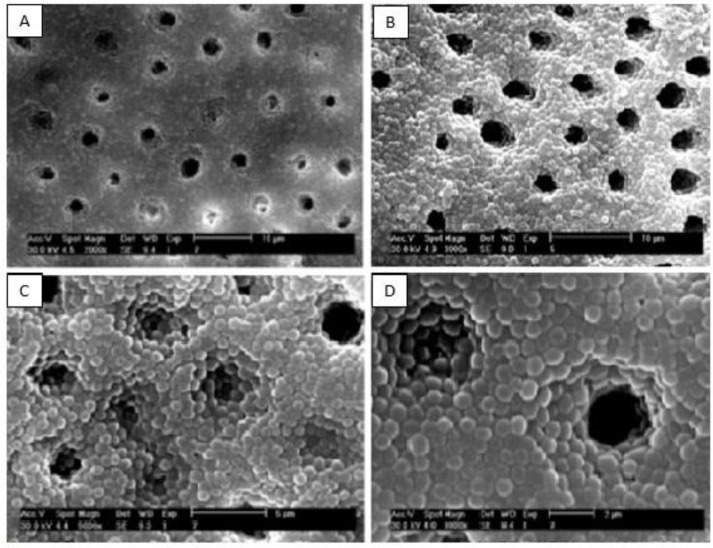
Scanning electron microscopy (SEM) images of the biofilm of Enterococcus faecalis (EF) bacterium formed on root canal walls and in dentinal tubules at (A) ×2000, (B) 3000, (C) 5000, and (D) 8000 magnifications

Distilled water was removed from the microtubes by the use of a sterile syringe. A suspension (0.5 McFarland) of *EF* (ATCC 29212) was prepared in TSB and was inoculated into the root canals of all the teeth, except for five teeth which were considered as negative controls. The teeth were immersed in sterile microtubes containing 1 ml of TSB, and then, they were incubated for a week at 37°C. The broth culture media were changed every other day. At the end of the incubation period, to confirm the formation of *EF* biofilm on root canal walls, four infected teeth were randomly selected, cut along their longitudinal axis, and examined by a scanning electron microscope (SEM; Zeiss DSM 960A, Carl Zeiss, Oberkochen, Germany; Fig. 1).

After the incubation period, the teeth were randomly divided into four groups:

Group 1: 24 teeth related to the TAP, which were divided into two subgroups (n=12).
Group 2: 24 teeth related to Ca(OH)_2_/2% CHX, which were divided into two subgroups (n=12).
Group 3: Positive control (five teeth which only contained bacteria).
Group 4: Negative control (five sterile teeth which were kept in sterile TSB to show the quality of apical and coronal seals).

The triple antibiotic powder was prepared by mixing equal amounts of Metronidazole (Parsdarou Co., Tehran, Iran), Ciprofloxacin (Razak Co., Tehran, Iran), and Doxycycline (Razak Co., Tehran, Iran). Then, a dose of 40 kGy of gamma radiation was used for sterilization of the powder. Afterwards, 4g of the triple antibiotic powder was weighed by Sartorius analytical balance (Sartorius BP211D, Sartorius Co., Göttingen, Germany) and was mixed with 4.5 ml of normal saline to achieve a favorable clinical consistency. Then, the prepared paste was transferred into the root canal of the 24 teeth in the first group by using a #2 lentulo spiral (DiaDent, Almere, the Netherlands). In the second group, 4g of Ca(OH)_2_ powder (Golchai Co., Tehran, Iran) was mixed with 4 ml of 2% CHX liquid (Calasept®, Directa Co., Upplands Väsby, Sweden) to achieve a favorable clinical consistency, the same as that of the TAP group, and then, the paste was transferred into the 24 root canals of the second group. Then, the coronal orifice of the root canals in both test groups was sealed with a thin layer of rose wax covered with a layer of nail varnish. The apical foramina of the root canals were also sealed with a layer of nail varnish to prevent the leaching of drugs from the root canals into the TSB containing the teeth. Positive control samples were sealed in the same way. Afterwards, all the teeth (groups 1, 2, 3, and 4) were placed in sterile microtubes containing 1.5 ml of sterile TSB and were kept at 37°C for a week. The TSB was refreshed every other day to prevent dehydration and to simulate the clinical condition. According to the pilot study, 12 teeth on day six and 12 teeth on day seven were evaluated after intracanal placement of the TAP. The teeth were removed from the microtubes under a biological hood and were placed in a sterile environment (the internal surface of a sterile surgical glove). The coronal seal was removed by the use of sterile forceps. The root canal space of each tooth was washed with 0.9% sterile normal saline, and then, it was dried with sterile paper cones. Finally, all the teeth were sectioned parallel to their longitudinal axes by using a diamond disk (DiaDent, Maribor, Slovenia). Dentin chips were obtained from the apical third of the root canals by means of a #04 round bur (Teeskavan Co., Tehran, Iran) and were collected in 0.5-ml microtubes.

According to the pilot study, in group 2 (Ca(OH)_2_/2% CHX), dentin chips were obtained from the apical third of the root canals of 12 teeth on the second day and of 12 teeth on the third day.

The powder obtained from each sample was weighed by the Sartorius analytical balance to give it an amount equal to that of the other samples so that a correct comparison between the groups could be made.

100 μl of sterile normal saline was added to each microtube containing the dentin chips. To determine the number of *EF* colonies, the serial dilution method with a 10-fold dilution was used. Finally, 50 μl of each diluted sample was inoculated into a TSB plate and was kept at 37°C for 24 hours, and the CFU/ml was calculated after about 24 hours. The above steps were repeated for both control groups. Data were collected and analyzed by using SPSS version 22 software (IBM Co., Chicago, IL, USA) via Wilcoxon signed-rank test.

## RESULTS

On the seventh day, the TAP eliminated the *EF* bacteria in all the dentinal samples obtained from the apical part of the root canals in group 1 (Table 3).

**Table 3.** Colony-forming unit (CFU)/ml of each study group at different time intervals in the main study

**Tooth number**	**TAP (group 1)**		**Ca(OH)_2_ (group 2)**	

	**Day 6**	**Day 7**	**Day 2**	**Day 3**
1	1.2×10^3^	0	0	0
2	1×10^3^	0	0	0
3	2.3×10^3^	0	1.14×10^4^	0
4	2.8×10^3^	0	0	0
5	1.7×10^3^	0	0	0
6	2.9×10^3^	0	0	0
7	1.1×10^3^	0	0	0
8	1×10^3^	0	8.1×10^3^	0
9	2.4×10^3^	0	0	0
10	1.8×10^3^	0	0	0
11	2.4×10^3^	0	1.25×10^3^	0
12	1.7×10^3^	0	0	0
Mean	1.85×10^3^	0	1.7×10^3^	0
Standard deviation (SD)	0.693×10^3^	0	3.828×10^3^	0
**P-value**	**P<0.001**		**P<0.001**	

TAP=Triple antibiotic paste, Ca(OH)_2_=Calcium hydroxide

In group 2, Ca(OH)_2_/2% CHX paste was able to significantly decrease the *EF* in most of the samples on the second day, but on the third day, this paste completely eliminated the *EF* in all the samples so the number of CFU/ml in all the specimens reached zero (Table 3).

At the end of the study, the teeth in groups 3 and 4 (positive and negative controls) were also evaluated. Table 4 shows the CFU/ml in group 3. No growth was observed in group 4.

**Table 4.** The results of Colony-forming unit (CFU) count in positive control group

**Tooth number**	**CFU**
1	4.72×10^4^
2	6.2×10^4^
3	5.64×10^4^
4	3.8×10^4^
5	4.26×10^4^
Mean	4.924×10^4^
Standard deviation (SD)	9.857×10^3^

## DISCUSSION

Various models have been proposed to evaluate the antimicrobial strength of ICMs. The model used in this study is a modified technique which was used by Haapasalo and Orstavik in 1987 [22]. Due to the significant difference in the canal’s diameter between human and bovine teeth, human permanent teeth were used in this study to make the conditions more similar to the clinical situation. In addition, with this model, it was possible to investigate the effect of antimicrobial agents on the biofilm form of the bacterium.

*EF* bacterium was used as target bacterium in this study as it is the most commonly isolated species in failed endodontic treatments and it is usually resistant to antimicrobial agents [2,4,7,29].

The TAP has been evaluated for its use in endodontic regeneration and in the treatment of resistant infections [30,31]. Regarding the use of this paste as an ICM, the mean duration for a TAP to exert its antimicrobial effect has been reported to have a range from 7 to 21 days [32,33]. Ca(OH)_2_ is one of the most common ICMs, which is also used in regenerative treatments [34,35]. The duration for this substance to exert its antimicrobial effect as an ICM has been reported to have a range from 24 hours to one week [10,15]. Many studies have shown that if Ca(OH)_2_ paste is prepared with 2% CHX, it will have an antibacterial effect significantly higher than that of Ca(OH)_2_ paste mixed with normal saline [12,17,19,36]. Therefore, in the present study, a mixture of Ca(OH)_2_ and 2% CHX was used as an ICM. In previous studies, no investigation has been done with regard to the shortest time required for a TAP and Ca(OH)_2_/2% CHX paste to completely eradicate the *EF* biofilm.

Therefore, the aim of this study was to determine the shortest time required for each of these two substances to reach the desired function, which is rendering a root canal system free of *EF* biofilm.

In a study by Evans et al [12], it was shown that a mixture of Ca(OH)_2_ and 2% CHX is more effective than Ca(OH)_2_/saline paste against EF after a week. They concluded that an additive or synergistic antimicrobial effect might result from the mixture of Ca(OH)_2_ and CHX [12].

In a study by Schafer and Bossmann [19], 2% CHX gel was more effective than Ca(OH)_2_ in combination with CHX or normal saline, and it was able to remove the *EF* biofilm after a three-day period. They also showed that the antibacterial strength of Ca(OH)_2_ mixed with CHX was higher than that of Ca(OH)_2_ mixed with saline, although the observed difference was not significant. The difference in the duration of the effect of Ca(OH)_2_ paste on *EF* biofilm between the study by Schafer and Bossmann [19] and this study could be due to the difference in the microorganism strains. The target microorganism was ATCC 6057 in the study by Schafer and Bossmann [19]. In the present study, the apical portion of root canals was used for collecting dentin chips, but in the cited study, the whole length of the canals was used [19]. After applying the medication, they used a new sterile Hedstrom file for canal preparation. Then, they transferred each file along with the dentin removed from the canal wall to a test tube. The instrument was then shaken in the test tube by a vibrator for 10 seconds, and finally, the CFU/ml in the shaken solution was determined by adopting standard laboratory methods. Therefore, the difference in collecting the samples could also have a role in the disagreement between the results of these two studies. In the study by Schafer and Bossmann [19], on the third day, Ca(OH)_2_/2% CHX paste did not completely eliminate the *EF* biofilm, but in the current study, the number of bacteria reached zero on the third day after intracanal placement of the paste.

According to the results of the current study, Ca(OH)_2_ in combination with 2% CHX was able to remove the target microorganism from the root canal system in a shorter period, while in the study by Adl et al [26], the TAP was the most effective agent in removing the *EF*. The difference between the results of this study and those of the mentioned study could be due to differences in their methods.

In the study by Adl et al [26], agar diffusion test was used to evaluate the antimicrobial activities of the medicaments, while in the present study, single-rooted human teeth and bacterial biofilm were used for this evaluation. In another study by Adl et al [32], the TAP was more effective than Ca(OH)_2_ in a seven-day period, and it eliminated *EF* from the root canal system, which was different from the results of the present study; however, it should be mentioned that in the cited study, saline was used in combination with Ca(OH)_2_.

Sato et al [21] examined the antibacterial effects of a TAP on Escherichia coli within the root canal system and concluded that it can remove all target bacteria from the root canal within 48 hours. In the present study, the target bacterium was *EF* and its standard strain of 29212 was selected. This difference in target bacteria could be one of the reasons for the difference between the present results and those of the study by Sato et al [21].

Also, in the present study, the effect of this paste on the biofilm form of the bacterium was evaluated, and the dentin chips in this study, unlike those in the study by Sato et al [21] (harvested from the surface of dentinal walls of the root canal by gates glidden drills), were obtained by a #04 round bur with a diameter of 400 μm and were later examined for microbial contamination. According to a study by Saber and El-Hady [37], antibiotics were more effective than Ca(OH)_2_ in removing the bacterial biofilm. In the present study, Antibiotics were able to remove the *EF* biofilm in a period of seven days, similar to the results of the study by Saber and El-Hady [37]. In the mentioned study, a Ca(OH)_2_ paste was prepared with saline, but in the present study, 2% CHX was used to prepare the Ca(OH)_2_ paste, which could be the reason for the higher antibacterial performance of this paste compared to the paste prepared with saline.

Based on an article by Ghabraei et al [38], the minimum time required by the TAP to eliminate the *EF* biofilm from the root canal system was seven days, which was similar to our results; it should be mentioned that the methods of the two studies were identical.

Although based on this study, Ca(OH)_2_/2% CHX paste was able to remove the *EF* biofilm from the root canal system after three days, it is not possible to generalize these results to the clinical situation. According to some studies, dentin, hydroxyapatite, and necrotic pulpal remnants can negatively affect the antibacterial activities of Ca(OH)_2_ [10].

Therefore, more thorough and precise studies are needed to determine the exact time required by Ca(OH)_2_ to remove the bacterial biofilm. This study only examined the effect of two ICMs on CFU count of EF in vitro. In this study, the Ca(OH)_2_/2% CHX paste was more effective than the TAP. Since the use of antibiotics, even topically, has led to concerns about bacterial resistance to ICMs, and since there is a possibility of discoloration due to Doxycycline in the composition of the TAP [28], Ca(OH)_2_/2% CHX paste may be considered as an alternative with fewer complications in regenerative treatments and in treatment-resistant infections.

## COUCLUSION

Ca(OH)_2_/2% CHX paste can eliminate the *EF* biofilm from root canal walls in a shorter time compared to a TAP. The minimum time required by Ca(OH)_2_/2% CHX paste and a TAP to eliminate *EF* from the root canal system is three days and seven days, respectively.
